# Effects of a Short-Term Recreational Team Handball-Based Programme on Physical Fitness and Cardiovascular and Metabolic Health of 33-55-Year-Old Men: A Pilot Study

**DOI:** 10.1155/2018/4109796

**Published:** 2018-10-03

**Authors:** Susana C. A. Póvoas, Carlo Castagna, Carlos Resende, Eduardo Filipe Coelho, Pedro Silva, Rute Santos, Rita Pereira, Peter Krustrup

**Affiliations:** ^1^Research Center in Sports Sciences, Health Sciences and Human Development, CIDESD, University Institute of Maia, ISMAI, Maia, Portugal; ^2^Fitness Training and Biomechanics Laboratory, Italian Football Federation, Technical Department, Coverciano, Florence, Italy; ^3^School of Sport and Exercise Sciences, University of Rome Tor Vergata, Rome, Italy; ^4^University Institute of Maia, ISMAI, Maia, Portugal; ^5^Centre of Research, Education, Innovation and Intervention in Sport, Faculty of Sport, University of Porto, Porto, Portugal; ^6^Portuguese Handball Federation, Portugal; ^7^Research Centre in Physical Activity, Health and Leisure, Faculty of Sport, University of Porto, Porto, Portugal; ^8^Early Start Research Institute, School of Education, Faculty of Social Sciences, University of Wollongong, Wollongong, Australia; ^9^Department of Sports Science and Clinical Biomechanics, Sport and Health Sciences Cluster (SHSC), University of Southern Denmark (SDU), Odense, Denmark; ^10^Sport and Health Sciences, University of Exeter, Exeter, UK

## Abstract

Recreational team handball is an intermittent high-intensity exercise mode with physiological demands in the range of those found to enhance health and physical fitness of sedentary adults. We examined the effects of a short-term team handball-based training programme on physical fitness and metabolic and cardiovascular health of sedentary 33-55-year-old former male team handball players. Twenty-four participants were divided into team handball (THG;* n*=15) and control groups (CG;* n*=9) and evaluated at baseline and postintervention. During 12 weeks, THG performed 2-3 60-min recreational team handball matches weekly (average: 2.2 ± 0.7), and CG maintained an inactive lifestyle. Average heart rate (HR) during matches was 80 ± 7%HR_max_, with peak values of 91 ± 6%HR_max_. A time-by-group interaction was shown in aerobic performance (*p*=0.016), postural balance (*p*=0.019), maximum oxygen uptake (VO_2_max) (*p*=0.023), resting HR (*p*<0.001), high-density lipoprotein (HDL) cholesterol (*p*=0.048), and fasting blood glucose (*p*=0.052) in favor of THG. THG improved aerobic performance (80%,* p*<0.001), VO_2_max (14%,* p*<0.001), and postural balance (27%,* p*=0.018). Decreases in resting HR (16%,* p*<0.001) and fasting blood glucose (7%,* p*=0.015) and increases in HDL cholesterol (11%,* p*=0.002) were found in THG. Recreational team handball practice shows positive physical fitness and health-related adaptations, with high attendance, which may contribute to the reduction of the risk of developing lifestyle diseases.

## 1. Introduction

In modern society, the adoption of sedentary lifestyles results in low physical fitness levels, one of the most important risk factors for chronic-degenerative diseases, especially those related to the cardiovascular system [1, 2]. Epidemiologists and public health promoters have focused on the detection and prevention of modifiable risk factors that are associated with these diseases, particularly, low cardiorespiratory fitness, through exercise interventions since exercise is associated with a decreased risk of cardiovascular and metabolic diseases [3, 4]. Despite the health benefits of regular exercise on health and physical fitness, only 8% of the European population exercises regularly, whereas as much as 20% indicates lack of motivation as one of the causes for not exercising more frequently [5]. Therefore, novel exercise modes stimulating behavioural changes in sedentary subjects are warranted from a public health perspective. Some team sports have been reported to constitute a sustainable training intervention, effective in inducing wide range health and fitness improvements, in a motivating environment, though research in this area is still scarce [6].

In the last decade, recreational soccer has led the way in the field of using team sports as an alternative exercise mode in the treatment and prevention of lifestyle chronic diseases [7]. Positive broad-spectrum effects have been reported for different populations in response to short- and long-term recreational soccer training programmes while showing high attendance rate and moderate adherence [6–8].

Recently, it was shown that recreational team handball is an intermittent high-intensity exercise mode, with high aerobic and anaerobic demands, similar to the competitive version [9]. Indeed, for the recreational team handball outfield players, mean heart rate (HR) values were 82%HR_max_, and for 71% of total match time (i.e., 60 min) the HR was above 80%HR_max_ and for 24% above 90%HR_max_ (42 and 14 min, respectively). The imposed cardiovascular strain is within the range of that considered sufficient to cause marked improvements in cardiorespiratory fitness, systolic blood pressure, and glucose tolerance, increasing the overall health profile [10–12]. The high-intensity nature of recreational team handball was reported to be the result of the jumps, throws, stops, changes of direction, one-on-one situations, and unorthodox movements performed throughout the matches [9]. Indeed, for 75% of the reported 60-min matches, the recreational team handball players were standing still or walking, covering a total of 6 km [9].

Recreational team handball match demands are in line with those described for recreational soccer practice [13] which has shown to positively impact on health and physical fitness of different populations [8, 14, 15]. Thus, recreational team handball can also potentially be considered as a useful exercise intervention for the development of cardiovascular and musculoskeletal fitness in adult males that are former handball players, consequently broadening the range of health and fitness beneficial exercise modes for the general population. Considering that recreational team handball playing could be an appealing exercise mode for a relevant number of former players and fans worldwide [16], training studies using this sport as a health and physical fitness enhancing intervention are warranted.

Therefore, the purpose of this study was to examine the effects of a short-term recreational team handball-based exercise programme on physical fitness and metabolic and cardiovascular health of sedentary 33-55-year-old male adults, formerly trained in this team sport. Practically relevant improvements in players' physical fitness and most of the health-related variables were assumed as the likely effect of the recreational team handball intervention (i.e., work hypothesis).

## 2. Materials and Methods

### 2.1. Subjects

Twenty-four participants were involved in this study (Table 1). Fifteen composed the team handball training group (THG: 13 outfield players and 2 goalkeepers) and 9 the control group (CG). All the participants had previous experience with the sport. The participants had played team handball for 15 ± 8 years and their training history was 2-7 weekly training sessions on average, performing a total of 7 ± 4 hours per week, though reporting no record of regular physical activity for the last 14 ± 6 years.

No significant differences were detected at baseline between the two groups in chronological age and in the anthropometric, physical fitness and cardiovascular and metabolic health variables, except for resting HR, with the CG participants showing higher values (*p*=0.005; Tables 1 and 2).

### 2.2. Experimental Design

The recreational team handball training intervention was held for 12 weeks. During this period, the participants in the THG performed 2-3 training sessions of 75-min per week, consisting of a standardized 10-min warm-up followed by 60 min of playing recreational team handball matches (6v6 and 7v7), interspersed by a 5-min half-break. The standardized warm-up consisted of 5 min of self-paced jogging, running at progressively increasing speeds, changes of direction, arm rotation with the ball, and 5 min of technical ball drills (passes, throws to goalie with a goalkeeper, and one-on-one situations). No time-out periods were allowed during the matches unless for players' physical assistance (e.g., injuries). The training sessions were held with, at least, a 48 h rest period in-between, in an indoor team handball court (40x20 m). Average training attendance was 2.2 ± 0.7 (0.7–3.0) sessions per week, corresponding to a total average of 26 ± 9 (8–36) sessions during the 12-week intervention period (i.e., 36 training sessions). The participants in the CG maintained their usual daily physical activity, i.e., inactive lifestyle, and both groups reported no changes in their diet during the 12-week period.

Physical fitness and health evaluations were performed at baseline and at the end of the 12 weeks. No physical activities were performed 2 days before the testing. Before the commencement of this study the participants were informed of the aims and the risks associated with the experiment, before delivering their informed written consent to participate, and familiarized with all testing protocols and procedures during specific sessions performed in the weeks before the starting of the study.

The experimental protocol was approved by the local Institutional Review Board and followed the Declaration of Helsinki.

### 2.3. Experimental Procedures

Stature was measured to the nearest millimeter in bare feet with the participants standing upright against a stadiometer (Holtain Ltd., Crymych, Pembrokeshire, UK). Body mass and percentage of fat mass were measured using a portable electronic scale (Tanita InnerScan digital BC532, Amsterdam, The Netherlands) with the participants wearing shorts. Body mass was measured to the nearest 0.1 kg. Resting HR and blood pressure—systolic blood pressure (SBP), diastolic blood pressure (DBP), and mean arterial pressure (MAP)—were measured by an automatic upper arm blood pressure monitor (multiparameter patient monitor, Omron Z207, Kyoto, Japan). The participants were required to sit and rest for at least 5 min prior to the first blood pressure measurement. Participants were in a seated, relaxed position with their feet resting flat on the ground. Two measurements were taken after 5 and 10 min of rest and the mean of these two measurements was considered for blood pressure analysis. If the two measurements differed by two mmHg or more, a third measure was taken. The lowest resting HR value was considered for analysis.

In order to determine plasma total cholesterol, high-density lipoprotein (HDL) and low-density lipoprotein (LDL) cholesterol, triglycerides, blood glucose, and plasma insulin values, venous blood samples were collected from the antecubital arm vein (left or right) using a standard operating procedure, in the morning (8-10 a.m.) and after an overnight fast of at least 8 hours. Blood markers were determined using automated analyzers—total cholesterol, HDL and LDL cholesterol, triglycerides (enzymatic color assay; Automated Olympus AU5400, Beckman-Coulter equipment, Brea, USA), glucose (UV enzymatic assay; Automated Olympus AU5400, Beckman-Coulter equipment, Brea, USA), and insulin (Electrochemiluminescence Immunoassay; Cobas e411, Roche Diagnostics Gmbh, Mannheim, Germany)—in a clinical laboratory.

Immediately after basal samples collection, the participants were asked to drink 0.2 L of a solution containing 75 g of glucose and after two hours a 3-ml venous blood sample was collected to measure plasma glucose and insulin.

To determine blood lactate concentrations ([Blac]), capillary blood samples (30 *μ*L) of fifteen players were drawn from the right earlobe: at rest; at 5, 10, 15, 20, and 25 min; and at the end of the first and of the second halves of the matches. A portable electroenzymatic lactate device analyzer (Lactate ProTM, Quesnel, Canada) was used.

The carotid-femoral pulse wave velocity (PWV), a marker of arterial stiffness, was assessed in a quiet, dimmed room by a single trained cardiopneumology technician with a portable device (Micro Medical®, model PulseTrace PWV PT4000, Kent, UK) with the participants placed in a supine position. After 5 min of rest, the carotid-femoral distance was assessed as the distance of suprasternal notch to the umbilicus and from there to the measuring point at the femoral artery plus the suprasternal notch to the measuring point at the carotid artery. Electrocardiogram registry was performed simultaneously, allowing the software to calculate the time from the peak of the R-wave to the foot of the pulse wave at the carotid and femoral arteries, respectively. The digital volume pulse waveform had to fill 2/3 of the display with little or no noise and artifact to be considered and three 10-seconds measurements of PWV were performed and averaged for analysis.

In order to determine maximal oxygen uptake (VO_2_max), the participants performed an incremental treadmill (Quasar-Med, Nussdorf, Germany) test until voluntary exhaustion [17]. Expired respiratory gas fractions were measured using an open circuit breath-by-breath automated gas-analysis system (Cortex, Metalyzer, 3B, Leipzig, Germany). Aerobic performance was assessed by the Yo-Yo intermittent endurance test–level 2 (YYIE2) [18]. HR_max_ was considered as the highest HR value achieved from the incremental treadmill test or the YYIE2 using short-range telemetry (Firstbeat Technologies Ltd., version 4.5.0.2, Jyväskylä, Finland).

A single-legged Flamingo balance test was used to evaluate postural balance control [19]. Subjects were instructed to stand on the dominant leg with their eyes open on a 3-cm wide and 5-cm high bar, while the free leg was flexed at the knee joint and held at the ankle joint close to the buttocks. The number of falls was counted during one minute of stance as a measure of postural balance. The participants had one trial and a 1-min period of familiarization was performed before all the tests.

Upper body isometric strength (handgrip strength test) was assessed using a handgrip dynamometer (T.K.K. 5401, Grip-D, Takei, Japan), adjusted by hand size for each participant. The participants were instructed to stand with their arms completely extended, squeezing gradually and continuously the handgrip up to the maximum of their strength, for at least 2 seconds. Participants performed the test twice in the dominant hand. A 90-s period rest was given between trials. The best score was recorded in kilograms [20].

The training sessions were monitored for match time-motion analysis (*n* = 44 records, 32 outfield players, 12 goalkeepers, 6 matches), HR (*n* = 194 records, 128 outfield players, 66 goalkeepers, 18 matches), blood lactate (*n* = 36 records, 36 outfield players, 10 goalkeepers, 6 matches), and rating of perceived exertion (RPE) for all matches. Experimental procedures regarding these analyses are described elsewhere [9]. Training sessions intensity testing was performed at the end of the afternoon, and the participants were advised to eat a normal diet, including carbohydrates, the day before testing and to eat lunch at least 2 hours before testing and to be properly hydrated.

### 2.4. Statistical Analyses

The results are presented as mean ± standard deviation (SD). The student's unpaired* t*-test was used to assess baseline and delta values differences in the selected variables between the groups after the 12 weeks. The differences between the groups at baseline and at postintervention were examined using a two-way analysis of variance (ANOVA) for repeated measures, with Bonferroni post hoc multiple comparison tests. Practical significance was assessed by calculating partial eta squared (*η*_ _^2^_p_) (values of 0.01, 0.06, and above 0.15 were interpreted as small, medium, and large, respectively) [21]. Pearson correlation was used to assess variables associations, and magnitude of effects was described using the Hopkins et al. [22] guidelines. Statistical Package for the Social Sciences (SPSS Inc., version 23.0) was used for all analyses. The data were tested for normality using the Shapiro-Wilk test. Statistical significance was set at* p*≤0.05.

## 3. Results

### 3.1. Training Sessions Intensity

During the team handball matches, which lasted on average 60 ± 3 min, the outfield players covered a total distance of ~6.5 km (6410 ± 416 m), of which ~1 km was covered with high-intensity movements (934 ± 426 m). Unorthodox movements, which equaled the sum of the distance covered during backwards, sideways medium-intensity and sideways high-intensity movements, accounted for 2% of the total distance covered (136 ± 162 m).

Changes of match activity occurred 392 ± 64 times on average per match, and the sum of high-intensity runs and unorthodox movements were 63 ± 16 and 28 ± 24 per match, respectively. Jumps (17 ± 11) and throws (16 ± 7) were the most frequent highly demanding playing actions. The outfield players performed an average of 5 ± 5 stops, 8 ± 6 changes of direction, and 9 ± 5 one-on-one situations per match.

For the outfield players, the mean HR during the matches was 145 ± 15 b^.^min^−1^ and peak HR was 165 ± 14 b^.^min^−1^ (81 ± 6 and 92 ± 5%HR_max_, respectively).

For 45% of total match time (27 ± 10 min) the outfield players' HR was between 81 and 90%HR_max_ and for 21% (13 ± 12 min) above 90%HR_max_. HRs were equal to or lower than 70%HR_max_ for only 14% of the match time (8 ± 5 min). Average and peak [Blac] were 3.7 ± 1.5 (1.3-6.8) mM and 4.4 ± 1.3 (2.7-6.8) mM, respectively. The outfield players' postmatch RPE was 7.3 ± 0.9 (2-10) arbitrary units (AU; very hard).

Jumps were the actions most frequently performed by the goalkeepers (37 ± 7), followed by changes of direction (28 ± 5), defense actions (22 ± 3), and stops (17 ± 7). Peak and mean HRs were 149 ± 29 and 127 ± 29 bpm (81 ± 6 and 73 ± 10%HR_max_), respectively. The goalkeepers' cardiovascular load was between 81 and 90%HR_max_ for 25% of the total match time and only 7% above 90%HR_max_, while HRs were equal to or below 70%HR_max_ for 40% of total match time. Average [Blac] was 1.1 ± 0.1 (0.9-1.2) mM and postmatch RPE was 6.3 ± 2.1 (1-9) AU (hard). Match demands were significantly different between goalkeepers and outfield players (*p*<0.050). When considering all participants in the THG (i.e., outfield and goalkeepers), the average HR during the matches was 80 ± 7%HR_max_, with peak values of 91 ± 6%HR_max_.

### 3.2. Physical Fitness

Although goalkeepers' training intensity differs from the outfield players, data from this playing position was included in the pre- versus post-physical fitness, cardiovascular and metabolic markers statistical analysis, since it did not relevantly impact the descriptive statistics or alter the results from the inferential analysis.

**Figure 1:**
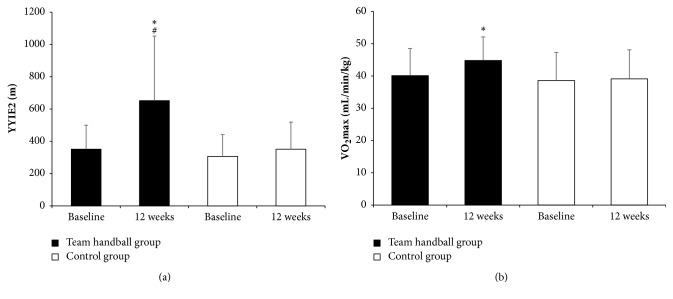
Yo-Yo intermittent endurance test, level 2 performance (YYIE2, Figure 1(a)) and maximal oxygen uptake (VO_2_max, Figure 1(b)) at baseline and after 12 weeks of recreational team handball practice (*n* = 15; team handball group) or a continuation of an inactive lifestyle (*n* = 9; control group). *∗* means significantly different from baseline (*p*≤0.05); # means significantly different from the control group (*p*≤0.05).

A time-by-group interaction was shown in YYIE2 performance (*p*=0.016; Figure 1(a)) and postural balance (*p*=0.019) with the THG showing a marked 80% (*p*<0.001) increase in YYIE2 at 12 weeks (351 to 652 m) and a 27% (*p*=0.018) drop in the number of falls in the postural balance test (16 to 11 falls; Table 2). At 12 weeks, the THG YYIE2 performance was almost twofold higher than that of the CG (652 ± 400 versus 351 ± 168 m;* p*=0.047) and the THG had half the falls in the balance test (11 versus 21,* p*=0.052).

### 3.3. Cardiovascular Health

**Figure 2:**
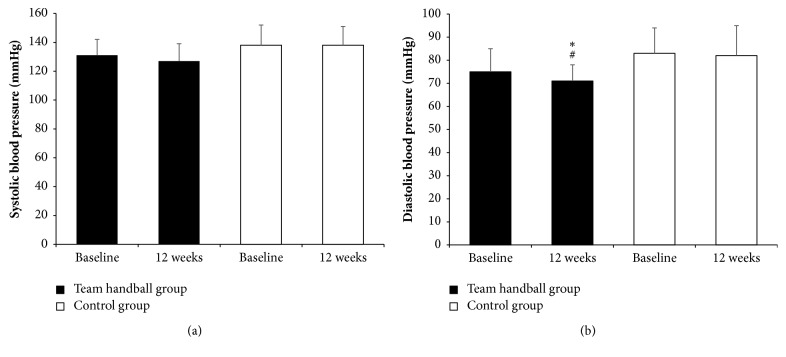
Systolic (a) and diastolic (b) blood pressure values at baseline and after 12 weeks of recreational team handball practice (*n* = 15; team handball group) or a continuation of an inactive lifestyle (*n* = 9; control group). *∗* means significantly different from baseline (*p*≤0.05); # means significantly different from the control group (*p*≤0.05).

A time-by-group interaction was shown in VO_2_max (*p*=0.023; Figure 1(b)) and resting HR (*p*<0.001) with the THG showing a 14% (*p*<0.001) increase in VO_2_max (40.1 to 44.8 mL/min/kg) and a 16% (*p*<0.001) decrease in resting HR (62 to 52 bpm) at 12 weeks (Table 2). No significant differences were found in SBP (Figure 2(a); Table 2). A group effect was shown in DBP (*p*=0.048; Figure 2(b); Table 2), with the THG showing an average 4 mmHg decrease at 12 weeks (75 to 71 mmHg) and values significantly lower than the CG (71 ± 7 versus 82 ± 13 mmHg;* p*=0.028; Table 2). A group effect was also shown in MAP (*p*=0.049). At 12 weeks, MAP was lower for the THG than for the CG (90 ± 8 versus 100 ± 12 mmHg;* p*=0.028).

### 3.4. Metabolic Health

A time-by-group interaction was shown in HDL cholesterol (*p*=0.048; Table 2) and fasting blood glucose (*p*=0.052) in favor of the THG. In this group, HDL cholesterol increased by 11% (*p*=0.002) at 12 weeks and fasting blood glucose decreased by 7% (*p*=0.015). A time effect was shown in total cholesterol, LDL cholesterol, total cholesterol/HDL ratio, total cholesterol/LDL ratio, and LDL/HDL ratio (*p*≤0.015; Table 2). Both groups showed a significant decrease in total cholesterol, LDL cholesterol, total cholesterol/HDL ratio, and LDL/HDL ratio (*p*≤0.030). Nonetheless, only the THG showed a significant increase in HDL cholesterol (*p*=0.002). The THG showed a medium to large significant decrement in fasting blood glucose, blood glucose, and plasma insulin after the two-hour oral glucose tolerance test (*p*≤0.015; Table 2).

## 4. Discussion

The present study examines, for the first time, the cardiovascular and metabolic health and physical fitness effects of short-term recreational team handball practice in sedentary adults (33–55 years) with previous experience with this sport. This training intervention showed that an average of 2.2 sessions per week of 60-min per day of recreational team handball practice, during 12 weeks, induced mainly moderate to large positive improvements in physical fitness and health-related variables in the participants. These findings confirmed this study work hypothesis and line up with the effects on fitness and health, previously demonstrated in training interventions using soccer, basketball, and floorball in similar populations [14, 23, 24].

Maximal oxygen uptake is considered a valid measure of cardiorespiratory and aerobic metabolism efficiency and a strong predictor of overall mortality [25, 26]. Thus, impairing the natural decrement of VO_2_max associated with aging is considered of importance in preventive medicine [27]. The recreational team handball intervention produced a large and significant 14% improvement in VO_2_max corresponding to approximately 5 mL/min/kg (i.e., ~1.43 METs). These results are of clinical significance as it is known that increments of 1 MET correspond to a 13% reduction in the risk of mortality [28, 29]. Our results are in line with other studies that used recreational soccer in the form of small-sided games carried out with populations of similar age to the participants in this study [14]. Indeed, Krustrup et al. [10] and Randers et al. [30] reported postintervention improvements in VO_2_max in the range of 8-13% as effect of 12 weeks of recreational soccer, performed ~2 times per week, in 20-43 year-old healthy untrained men. It has been suggested that the time spent at high-intensity during the recreational soccer matches, considered as match HRs above 90% of HR_max_, was the possible cause of the reported similar improvements in VO_2_max [14]. Furthermore, studies carried out with professional soccer players showed that time spent at intensities that induced HRs higher than 90%HR_max_ resulted in moderate to large improvement in aerobic fitness [31, 32]. Interestingly, in this study, the postintervention changes in VO_2_max were largely correlated with the time spent with HRs >90%HR_max_ (r=0.606,* p*=0.048). In fact, during the intervention the outfield players spent 21% or their match time with HRs >90%HR_max_, a percentage in the upper range of that reported for enhancing VO_2_max in recreational soccer interventions (12-30%) [14]. The reported improvement in VO_2_max was higher (i.e., 4.7 versus 2.4 mL/min/kg; 14% versus 3-4%) than that reported by Randers et al. [23] in untrained recreational younger (i.e., 28 years) basketball players that trained for similar time extension and weekly training frequency. The encouraging large improvement in VO_2_max found in this study may be due to the nature of team handball that, differently from soccer, involves a remarkable use of the upper limb muscles beside the legs [33]. This comparison may suggest the use of larger scale studies to understand whether there are additional advantages related to team sports involving arm and legs (team handball, basketball, and floorball) compared to those that mainly use lower limbs such as soccer.

A drop in resting HR has been reported as a reflection of functional adaptations in the autonomic nervous system induced by aerobic fitness improvements [34]. Cooney et al. [35, 36] reported that increases in resting HR per 15 bpm units likely augment the risk in incurring in cardiovascular disease by 24% in men. The THG reported a large, 16%, postintervention decrement in resting HR, suggesting a remarkable effect on the autonomic nervous system of recreational team handball practice. The raw change (i.e., -10 bpm) in resting HR of team handball practice was similar to that reported by Randers et al. [30] (-7 bpm) in healthy individuals, Schmidt et al. [37] in untrained elderly, and Andersen et al. [38] in mild hypertensive individuals (i.e., -8 bpm) participating in recreational soccer interventions over longer periods (i.e., 4-12 months).

The 12-week period of recreational team handball practice was successful in producing a remarkable 80% change (i.e., 302 m) in players' YYIE2 performance. The achieved change in YYIE2 performance was largely higher than those reported in recreational soccer studies (i.e., 37 to 49%) after similar intervention durations and addressing different populations [14]. Interestingly, the posttraining performance in YYIE2 was similar to baseline values in younger untrained individuals (i.e., 30 versus 42 years), despite trivial differences in baseline VO_2_max (40.1 ± 8.4 versus 40.0 ± 6.2 mL/min/kg) [39]. However, Krustrup et al. [39] reported that YYIE2 performance in untrained individuals was also dependent on anaerobic metabolism and this may explain the huge difference in YYIE2 performance despite similar VO_2_max values between the studies. The YYIE2 performance was reported to be largely associated with individual VO_2_max level in untrained individuals, although the test heavily stresses the anaerobic pathway [39]. Globally speaking, differences in baseline neuromuscular fitness, aerobic/anaerobic fitness, and age may have accounted for the reported differences in YYIE2 from this and other team sports studies. These results (i.e., VO_2_max, resting HR, and YYIE2 large improvements) provide evidence to support the effectiveness of recreational team handball practice in improving individual cardiovascular and anaerobic fitness. Given the interest of the possible cause-effect relationship between recreational team handball and physical fitness, future mechanistic training studies are warranted.

Recreational team sports interventions in the form of soccer, floorball, and basketball played as small-sided games were reported to induce significant changes in health-related variables, such as blood pressure and blood profile [8, 23, 24]. In this study, the THG experienced a 3%, 4%, and 4% postintervention decrement in SBP, DBP, and MAP, respectively. This finding indicates that team handball recreational practice positively affected the cardiovascular system of the participants. The 4 mmHg reduction in DBP was lower than the drop reported in untrained participants that volunteered in soccer, floorball, and street basketball training studies (5-8 mmHg) [14, 23, 24]. A comparison with floorball and basketball interventions shows results of interest, since, like in team handball, there is a significant use of upper limbs during match activities. In a street basketball training study, Randers et al. [23] showed changes in DBP that were not different from the considered control group, irrespective of the playing court length considered (i.e., full or half court). These results may suggest an effect of recreational team sports exercise mode on DBP changes, with small-sided soccer providing greater effects. The significant MAP decrease in the THG (i.e., 4 mmHg) was in line with the changes reported in street basketball (i.e., 5.6 mmHg) when players trained 3v3 on a full court [23]. It is interesting to note that when half court was considered (10x12 m versus 20x12 m) no changes in MAP were reported as effect of training, suggesting an effect of court size when considering team sport with remarkable use of the upper body during match activities. The team handball training programme resulted in practically small changes in SBP as previously reported in male recreational soccer players in similar age groups [8] and similar to those found by Randers et al. [23] at the end of a 3v3 basketball intervention when the players used half court. Decrements in blood pressure are usually associated with improved aerobic fitness and highly dependent on participants' baseline values [40]. Although not possible to demonstrate with this research design, the reported practically small changes in SBP in THG may have been the result of the training load (i.e., volume x intensity) experienced by THG during the 12 weeks. Further studies addressing this interesting health-related issue using longer intervention duration and more frequent weekly sessions of recreational team handball programmes are warranted.

Regular aerobic exercise has been shown to positively affect blood profile by lowering circulating cholesterol [41]. Total cholesterol was reported to follow an age dependent increase mainly until the age of 50 years [42]. However, a systematic review reported an age independent concentration of blood lipids and a significant effect of aerobic exercise on fasting total cholesterol and HDL and LDL cholesterol concentrations [43]. The recreational team handball training intervention proved effective in lowering some metabolic risk factors shown to induce cardiac dysfunctions, arteriosclerosis, and type II diabetes and to cause sudden mortality [41–43]. Indeed, the THG reported a large posttraining increase in HDL (i.e., +11%) and decrease in LDL (i.e., -14%) cholesterol. This was parallel to a large decrease in total cholesterol (i.e., -10%) and triglycerides (i.e., -15%). Variations in LDL cholesterol were reported to be mainly dependent on exercise induced body composition changes, while HDL cholesterol variations were attributed to training intensity [44]. The reported large practical changes in LDL values found in this study are in the upper range of those reported in recreational soccer studies that reported changes in LDL cholesterol (4-15%) [8, 14]. However, the changes in total cholesterol levels alongside the increments in HDL cholesterol achieved by the THG were superior to those found in recreational soccer [8, 14].

The PWV constitutes an indirect method to assess arterial stiffness considered as a variable which reflects alterations of vessels structure and function [45]. Upregulation of PWV is associated with cardiovascular risk factors such as hypertension and related to insulin resistance [46]. The practical medium decrement (i.e., -11%) in PWV found in the THG and associated improvements in blood pressure variables provide evidence of the beneficial effect of team handball practice on peripheral circulation and, thus, cardiovascular health.

Blood glucose concentration was considered as an indirect marker of insulin resistance and affected by aerobic and resistance exercise [47, 48]. The THG showed a large (i.e., -7%) decrement in fasting blood glucose, a finding different from that reported in recreational soccer and street basketball training interventions [8, 23]. Large positive changes were also observed in blood glucose and plasma insulin after the two-hour oral glucose tolerance test indicating improved insulin sensitivity. This finding may suggest an impact of diabetes II-related markers in THG after training. Given the importance of this issue in the quest for new exercise strategies to contrast type II diabetes pandemic, further studies are warranted.

Postural balance is considered a physical ability related to neuromuscular health and associated with the risk of falling [49, 50]. Recreational soccer was reported to provide practically relevant improvements in postural balance assessed by the Flamingo balance test [8]. The balance performance improvements achieved by the recreational soccer players ranged between +41 and 49%, remarkably higher than those of the THG players obtained following this study protocol (i.e., 27%). The large improvement in the Flamingo balance test found in this study provides evidence of the beneficial effect of team handball on fall prevention [49, 50]. In this regard, further studies carried out with the aim of examining the effect of recreational team handball in populations prone to falls, like the elderly, are warranted.

Handgrip strength has been described as a general maker of body strength and its reduced values have been reported to be associated with increased risk of cardiovascular and all-cause mortality across age and gender, in socially and culturally diverse populations [51]. Team handball requiring a solid ball grip to catch and pass the ball during the game was supposed to affect recreational team handball players' handgrip strength during the proposed intervention [9]. Results showed a practical small (i.e., +3%) improvement in handgrip strength postintervention in the THG participants. It could be speculated that the former competitive level status of the players in the THG may have had an effect on the magnitude of the reported changes in handgrip strength. Interestingly, although not significant, the reported changes in handgrip strength paralleled those related to cardiovascular and metabolic health, suggesting the potential of this simple measure to reflect general wellness [51]. Given the importance of this issue for large population studies, further investigations are warranted.

The changes shown in most of the physical fitness and metabolic and cardiovascular variables were similar between outfield players and goalkeepers, indicating that if the participants are previously trained in team handball, the beneficial effects may occur, regardless of the playing position. Nevertheless, one can speculate that if the participants are not specifically trained as goalkeepers, the demands would be quite lower due to lack of expertise [9, 52–54]. Thus, training interventions aimed at participants with no experience with this sport should rotate the players between these two playing positions in order to promote exercise intensity.

The CG showed no significant changes in all of the evaluated variables, with the exception of cholesterol (total and LDL), the ratios: total cholesterol/HDL, total cholesterol/LDL, and LDL/ HDL, and fasting plasma insulin, underlying the effectiveness of recreation team handball as a training intervention exercise mode.

In summary, short-term recreational team handball played as small-sided or formal games (i.e., 6v6 versus 7v7) is a high attendance team sport for 33-55-year-old sedentary former team handball players, which positively impacts on participants' physical fitness and cardiovascular and metabolic health. Training interventions aimed at analyzing the effects of recreational team handball practice on participants of either gender with little or no experience in this sport and also the physical and physiological demands of playing different game formats are warranted.

## Figures and Tables

**Table 1:** Chronological age and anthropometric characteristics (means ± SD) of the participants in the recreational team handball practice group (*n* = 15; team handball group) and the continuation of an inactive lifestyle group (*n* = 9; control group).

Variable	Team handball group (n=15)	Control group (n=9)
Age (years)	42.3 ± 7.1	40.2 ± 5.3
Height (cm)	179.4 ± 7.3	178.6 ± 4.3
Body Mass (kg)	97.5 ± 9.5	96.4 ± 20.2
Fat Mass (%)	22.9 ± 5.9	24.5 ± 7.6

**Table 2:** Physical fitness, cardiovascular and metabolic health markers at baseline and after 12 weeks of recreational team handball practice (*n* = 15; team handball group) or a continuation of an inactive lifestyle (*n* = 9; control group).

	**Team handball group (*n* = 15)**				**Control group (*n* = 9)**				**Two-way ANOVA**			*η*_ _^2^_*p*_	*p*
	Baseline	12 weeks	△ (abs)	△ (%)	Baseline	12 weeks	△ (abs)	△ (%)	Time (F; *p*)	Group (F; *p*)	Interaction (F; *p*)	Time; group; interaction	Time; group; interaction
**Physical Fitness**													
YYIE2 (m)	351±149	652±400^*∗*^#	302±277#	79.9±54.3#	307±134	351±168	44±103	17.4±37.5	12.629;	3.083;	6.973;	0.387; 0.134;	0.002; 0.094;
									0.002	0.094	0.016	0.259	0.016
Postural balance (*n*)	16±9	11±4^*∗*^#	−6±7#	−26.9±40.7#	18±11	21±15	3±7.0	11.6±34.8	1.012;	1.746;	6.710;	0.056; 0.093;	0.329; 0.204;
									0.329	0.204	0.019	0.283	0.019
Handgrip strength (kg)	53.7±8.1	54.9±7.0	1.2±4.9	3.1±10.2	52.3±9.9	49.6±7.6	−2.8±8.1	−3.9±14.4	0.268;	0.931;	1.836;	0.016; 0.052;	0.611; 0.348;
									0.611	0.348	0.193	0.097	0.193

**Cardiovascular health**													
VO_2_max (mL/min/kg)	40.1±8.4	44.8±7.3^*∗*^	4.7±4.1#	13.5±13.6#	38.6±8.7	39.1±9.0	0.6±1.7	1.5±5.2	10.291;	0.867;	6.290;	0.377; 0.049;	0.005; 0.365;
									0.005	0.365	0.023	0.270	0.023
Resting heart rate (bpm)	62±8#	52±6^*∗*^#	−10±7#	−15.5±9.5#	75±11	78±12	3±8	4.1±9.9	5.425;	27.581;	17.821;	0.222; 0.592;	0.031; <0.001;
									0.031	<0.001	<0.001	0.484	<0.001
Systolic blood pressure (mmHg)	131±11	127±12	−4±8	−2.8±6.0	138±14	138±13	0±15	0.8±10.5	0.448;	3.274;	0.669;	0.024; 0.154;	0.512; 0.087;
									0.512	0.087	0.424	0.036	0.424
Diastolic blood pressure (mmHg)	75±10	71±7^*∗*^#	−4±5	−4.3±6.3	83±11	82±13	−1±6	−1.6±7.7	3.466;	4.497;	0.808;	0.161; 0.200;	0.079; 0.048;
									0.079	0.048	0.381	0.043	0.381
Mean arterial pressure (mmHg)	93±10	90±8#	−4±5	−3.7±5.3	101±12	100±12	−1±8	−0.6±7.6	2.154;	4.448;	0.979;	0.107; 0.198;	0.159; 0.049;
									0.159	0.049	0.336	0.052	0.336
Arterial stiffness (PWV) (m/seg)	10.0±4.2	8.5±2.7	−1.5±3.2	−11.1±18.7	8.2±2.9	7.9±1.9	−0.4±1.1	−1.5±13.1	0.895;	0.361;	0.319;	0.069; 0.029;	0.363; 0.559;
									0.363	0.559	0.582	0.026	0.582

**Metabolic health**													
Total cholesterol (mmol/L)	5.6±1.3	4.9±0.7^*∗*^	−0.7±0.9	−10.3±15.6	5.9±0.8	5.3±0.9^*∗*^	−0.6±0.5	−10.5±8.5	15.095;	0.694;	0.077;	0.470; 0.039;	0.001; 0.416;
									0.001	0.416	0.785	0.005	0.785
HDL cholesterol (mmol/L)	1.1±0.2	1.3±0.1^*∗*^	0.1±0.1#	10.7±11.2#	1.2±0.2	1.2±0.3	0.0±0.1	0.6±8.7	7.324;	0.002;	4.530;	0.301; <0.001;	0.015; 0.966;
									0.015	0.966	0.048	0.210	0.048
LDL cholesterol (mmol/L)	3.9±1.2	3.2±0.6^*∗*^	−0.7±0.8	−14.2±23.4	4.2±0.7	3.5±0.8^*∗*^	−0.7±0.5	−17.1±12.5	22.213;	0.511;	0.016;	0.566; 0.029;	<0.001; 0.484;
									<0.001	0.484	0.900	0.001	0.900
Total cholesterol/HDL ratio	4.94±0.92	3.96±0.67^*∗*^	−0.98±0.58	−19.3±8.5	5.08±1.15	4.62±1.45^*∗*^	−0.46±0.48	−10.5±10.4	33.185;	0.738;	4.241;	0.661; 0.042;	<0.001; 0.402;
									<0.001	0.402	0.055	0.200	0.055
Total cholesterol/LDL ratio	1.50±0.26	1.58±0.16	0.08±0.15	6.4±8.6	1.44±0.09	1.56±0.15^*∗*^	0.13±0.10	8.8±6.9	10.698;	0.270;	0.562;	0.386; 0.016;	0.005; 0.610;
									0.005	0.610	0.464	0.032	0.464
LDL/HDL ratio	3.39±0.90	2.54±0.53^*∗*^	−0.86±0.56	−23.3±13.3	3.57±0.96	3.03±1.19^*∗*^	−0.54±0.41	−17.2±13.2	35.892;	0.721;	1.849;	0.679; 0.041;	<0.001; 0.408;
									<0.001	0.408	0.192	0.098	0.192
Triglycerides (mmol/L)	1.3±0.5	1.1±0.7	−0.2±0.4	−15.3±25.7#	1.3±0.7	1.4±0.8	0.2±0.4	15.2±26.5	0.003;	0.259;	3.495;	<0.001; 0.015;	0.960; 0.617;
									0.960	0.617	0.079	0.171	0.079
Fasting blood glucose (mmol/L)	4.7±0.5	4.4±0.4^*∗*^#	−0.3±0.4#	−6.6±8.2	4.9±0.3	5.0±0.6	0.1±0.4	1.1±8.2	2.016;	3.638;	4.359;	0.106; 0.176;	0.174; 0.074;
									0.174	0.074	0.052	0.204	0.052
Two-hour OGTT blood glucose (mmol/L)	5.6±1.3	4.5±0.7^*∗*^	−1.1±1.4	−17.0±20.0	5.3±1.2	5.5±1.3	0.1±1.3	4.0±26.3	2.530;	0.581;	3.847;	0.130; 0.033;	0.130; 0.456;
									0.130	0.456	0.066	0.185	0.066
Fasting plasma insulin (*μ*mol/L)	15±5	10±6	−5±5	−33.6±26.0	30±29	14±18^*∗*^	−15±25	−46.4±24.5	7.066;	2.072;	1.813;	0.294; 0.109;	0.017; 0.168;
									0.017	0.168	0.196	0.096	0.196
Two-hour OGTT plasma insulin (*μ*mol/L)	139±110	44±34^*∗*^	−95±115	−49.9±56.4	109±152	85±116	−24±55	9.4±95.4	7.202;	0.016;	2.526;	0.298; 0.001;	0.016; 0.902;
									0.016	0.902	0.130	0.129	0.130

Data is presented as mean ± sd.

^*∗*^Significantly different from baseline (*p*≤0.05).

^#^Significantly different from the control group (*p*≤0.05).

YYIE2: Yo-Yo intermittent endurance level 2 test; VO_2_max: maximal oxygen uptake; PWV: pulse wave velocity; HDL: high-density lipoprotein; LDL: low-density lipoprotein; OGTT: oral glucose tolerance test.

## Data Availability

The data used to support the findings of this study are available from the corresponding author upon reasonable request.

## References

[1] Booth F. W., Roberts C. K., Thyfault J. P., Ruegsegger G. N., Toedebusch R. G. (2017). Role of inactivity in chronic diseases: Evolutionary insight and pathophysiological mechanisms. *Physiological Reviews*.

[2] Thorp A. A., Owen N., Neuhaus M., Dunstan D. W. (2011). Sedentary behaviors and subsequent health outcomes in adults: a systematic review of longitudinal studies, 1996–2011. *American Journal of Preventive Medicine*.

[3] Halbert J. A., Silagy C. A., Finucane P., Withers R. T., Hamdorf P. A., Andrews G. R. (1997). The effectiveness of exercise training in lowering blood pressure: a meta-analysis of randomised controlled trials of 4 weeks or longer. *Journal of Human Hypertension*.

[4] Jeon C. Y., Lokken R. P., Hu F. B., van Dam R. M. (2007). Physical activity of moderate intensity and risk of type 2 diabetes: a systematic review. *Diabetes Care*.

[5] European Commission (2014). *Eurobarometer 412 - Sport and Physical Activity report 2014*.

[6] Fløtum L. A., Ottesen L. S., Krustrup P., Mohr M. (2016). Evaluating a Nationwide Recreational Football Intervention: Recruitment, Attendance, Adherence, Exercise Intensity, and Health Effects. *BioMed Research International*.

[7] Krustrup P., Aagaard P., Nybo L., Petersen J., Mohr M., Bangsbo J. (2010). Recreational football as a health promoting activity: a topical review. *Scandinavian Journal of Medicine & Science in Sports*.

[8] Bangsbo J., Hansen P. R., Dvorak J., Krustrup P. (2015). Recreational football for disease prevention and treatment in untrained men: A narrative review examining cardiovascular health, lipid profile, body composition, muscle strength and functional capacity. *British Journal of Sports Medicine*.

[9] Póvoas S. C. A., Castagna C., Resende C. (2017). Physical and Physiological Demands of Recreational Team Handball for Adult Untrained Men. *BioMed Research International*.

[10] Krustrup P., Nielsen J. J., Krustrup B. R. (2009). Recreational soccer is an effective health-promoting activity for untrained men. *British Journal of Sports Medicine*.

[11] Krustrup P., Dvorak J., Junge A., Bangsbo J. (2010). Executive summary: the health and fitness benefits of regular participation in small-sided football games. *Scandinavian Journal of Medicine & Science in Sports*.

[12] Nybo L., Sundstrup E., Jakobsen M. D. (2010). High-intensity training versus traditional exercise interventions for promoting health. *Medicine & Science in Sports & Exercise*.

[13] Randers M. B., Nielsen J. J., Bangsbo J., Krustrup P. (2014). Physiological response and activity profile in recreational small-sided football: No effect of the number of players. *Scandinavian Journal of Medicine & Science in Sports*.

[14] Milanović Z., Pantelić S., Čović N., Sporiš G., Krustrup P. (2015). Is Recreational Soccer Effective for Improving VO2max? A Systematic Review and Meta-Analysis. *Sports Medicine*.

[15] Milanović Z., Pantelić S., Sporiš G., Mohr M., Krustrup P. (2015). Health-Related Physical Fitness in Healthy Untrained Men: Effects on VO2max, Jump Performance and Flexibility of Soccer and Moderate-Intensity Continuous Running. *PLoS ONE*.

[16] Karcher C., Buchheit M. (2014). On-court demands of elite handball, with special reference to playing positions. *Sports Medicine*.

[17] Noakes T. D. (1988). Implications of exercise testing for prediction of athletic performance: a contemporary perspective. *Medicine & Science in Sports & Exercise*.

[18] Bangsbo J. (1994). *Fitness Training in Football: A Scientific Approach*.

[19] Deforche B., Lefevre J., De Bourdeaudhuij I., Hills A. P., Duquet W., Bouckaert J. (2003). Physical fitness and physical activity in obese and nonobese Flemish youth. *Obesity Research*.

[20] Ruiz J. R., Castro-Piñero J., España-Romero V. (2011). Field-based fitness assessment in young people: the ALPHA health-related fitness test battery for children and adolescents. *British Journal of Sports Medicine*.

[21] Levine T. R., Hullett C. R. (2002). Eta Squared, Partial Eta Squared, and Misreporting of Effect Size in Communication Research. *Human Communication Research*.

[22] Hopkins W. G., Marshall S. W., Batterham A. M., Hanin J. (2009). Progressive statistics for studies in sports medicine and exercise science. *Medicine & Science in Sports & Exercise*.

[23] Randers M. B., Hagman M., Brix J. (2017). Effects of 3 months of full-court and half-court street basketball training on health profile in untrained men. *Journal of Sport and Health Science*.

[24] Vorup J., Pedersen M. T., Melcher P. S., Dreier R., Bangsbo J. (2017). Effect of floorball training on blood lipids, body composition, muscle strength, and functional capacity of elderly men. *Scandinavian Journal of Medicine & Science in Sports*.

[25] Laukkanen J. A., Lakka T. A., Rauramaa R. (2001). Cardiovascular fitness as a predictor of mortality in men. *JAMA Internal Medicine*.

[26] Lee C. D., Blair S. N., Jackson A. S. (1999). Cardiorespiratory fitness, body composition, and all-cause and cardiovascular disease mortality in men. *American Journal of Clinical Nutrition*.

[27] Hawkins S. A., Wiswell R. A. (2003). Rate and Mechanism of Maximal Oxygen Consumption Decline with Aging. *Sports Medicine*.

[28] Kodama S., Saito K., Tanaka S. (2009). Cardiorespiratory fitness as a quantitative predictor of all-cause mortality and cardiovascular events in healthy men and women: a meta-analysis. *The Journal of the American Medical Association*.

[29] Blair S. N., Kohl H. W., Barlow C. E., Paffenbarger R. S., Gibbons L. W., Macera C. A. (1995). Changes in physical fitness and all-cause mortality: a prospective study of healthy and unhealthy men. *Journal of the American Medical Association*.

[30] Randers M. B., Nielsen J. J., Krustrup B. R. (2010). Positive performance and health effects of a football training program over 12 weeks can be maintained over a 1-year period with reduced training frequency. *Scandinavian journal of medicine & science in sports*.

[31] Castagna C., Impellizzeri F. M., Chaouachi A., Bordon C., Manzi V. (2011). Effect of training intensity distribution on aerobic fitness variables in elite soccer players: A case study. *The Journal of Strength and Conditioning Research*.

[32] Castagna C., Impellizzeri F. M., Chaouachi A., Manzi V. (2013). Preseason variations in aerobic fitness and performance in elite-standard soccer players: A team study. *The Journal of Strength and Conditioning Research*.

[33] Cardoso Marques M. A., González-Badillo J. J. (2006). In-season resistance training and detraining in professional team handball players. *The Journal of Strength and Conditioning Research*.

[34] Carter J. B., Banister E. W., Blaber A. P. (2003). Effect of endurance exercise on autonomic control of heart rate. *Sports Medicine*.

[35] Cooney M. T., Vartiainen E., Laatikainen T., Joulevi A., Dudina A., Graham I. (2010). Simplifying cardiovascular risk estimation using resting heart rate. *European Heart Journal*.

[36] Cooney M. T., Vartiainen E., Laakitainen T., Juolevi A., Dudina A., Graham I. M. (2010). Elevated resting heart rate is an independent risk factor for cardiovascular disease in healthy men and women. *American Heart Journal*.

[37] Schmidt J. F., Hansen P. R., Andersen T. R. (2014). Cardiovascular adaptations to 4 and 12 months of football or strength training in 65- to 75-year-old untrained men. *Scandinavian Journal of Medicine & Science in Sports*.

[38] Andersen L. J., Randers M. B., Hansen P. R. (2014). Structural and functional cardiac adaptations to 6 months of football training in untrained hypertensive men. *Scandinavian Journal of Medicine & Science in Sports*.

[39] Krustrup P., Bradley P. S., Christensen J. F. (2015). The Yo-Yo IE2 test: Physiological response for untrained men versus trained soccer players. *Medicine & Science in Sports & Exercise*.

[40] Cornelissen V. A., Smart N. A. (2013). Exercise training for blood pressure: a systematic review and meta-analysis. *Journal of the American Heart Association*.

[41] Wang Y., Xu D. (2017). Effects of aerobic exercise on lipids and lipoproteins. *Lipids in Health and Disease*.

[42] Ferrara A., Barrett-Connor E., Shan J. (1997). Total, LDL, and HDL cholesterol decrease with age in older men and women: The Rancho Bernardo Study 1984-1994. *Circulation*.

[43] Durstine J. L., Grandjean P. W., Davis P. G., Ferguson M. A., Alderson N. L., DuBose K. D. (2001). Blood lipid and lipoprotein adaptations to exercise. *Sports Medicine*.

[44] Durstine J. L., Grandjean P. W., Cox C. A., Thompson P. D. (2002). Lipids, lipoproteins, and exercise. *Journal of Cardiopulmonary Rehabilitation and Prevention*.

[45] Vlachopoulos C., Aznaouridis K., Stefanadis C. (2010). Prediction of cardiovascular events and all-cause mortality with arterial stiffness. A systematic review and meta-analysis. *Journal of the American College of Cardiology*.

[46] Urbina E. M., Gao Z., Khoury P. R., Martin L. J., Dolan L. M. (2012). Insulin resistance and arterial stiffness in healthy adolescents and young adults. *Diabetologia*.

[47] Ryan A. S. (2000). Insulin resistance with aging—effects of diet and exercise. *Sports Medicine*.

[48] Lin X., Zhang X., Guo J. (2015). Effects of exercise training on cardiorespiratory fitness and biomarkers of cardiometabolic health: A systematic review and meta-analysis of randomized controlled trials. *Journal of the American Heart Association*.

[49] Liu-Ambrose T., Nagamatsu L. S., Hsu C. L., Bolandzadeh N. (2013). Emerging concept: Central benefit model of exercise in falls prevention. *British Journal of Sports Medicine*.

[50] Sherrington C., Henschke N. (2013). Why does exercise reduce falls in older people? Unrecognised contributions to motor control and cognition?. *British Journal of Sports Medicine*.

[51] Leong D. P., Teo K. K., Rangarajan S. (2015). Prognostic value of grip strength: Findings from the Prospective Urban Rural Epidemiology (PURE) study. *The Lancet*.

[52] Póvoas S. C. A., Ascensão A. A. M. R., Magalhães J. (2014). Analysis of fatigue development during elite male handball matches. *The Journal of Strength and Conditioning Research*.

[53] Póvoas S. C. A., Ascensão A. A. M. R., Magalhães J. (2014). Physiological demands of elite team handball with special reference to playing position. *The Journal of Strength and Conditioning Research*.

[54] Póvoas S. C. A., Seabra A. F. T., Ascensão A. A. M. R., Magalhães J., Soares J. M. C., Rebelo A. N. C. (2012). Physical and physiological demands of elite team handball. *The Journal of Strength and Conditioning Research*.

